# Testosterone replacement therapy and aneurysm biology: current evidence and clinical implications

**DOI:** 10.1007/s12055-026-02250-4

**Published:** 2026-07-30

**Authors:** Helbert de Oliveira Manduca Palmiero, Eberval Gadelha Figueiredo

**Affiliations:** Division of Neurosurgery, University of São Paulo Medical School, Dr. Enéas de Carvalho Aguiar Ave., , São Paulo, Room5083, 25505403-000 Brazil

**Keywords:** Testosterone Replacement Therapy, Aneurysm Formation, Intracranial Aneurysm, Abdominal Aortic Aneurysm, Vascular Remodeling, Inflammation

## Abstract

**Background:**

Testosterone replacement therapy (TRT) is increasingly used to treat male hypogonadism, but its role in aneurysm biology remains unclear. Because aneurysm formation is driven by inflammation, extracellular matrix degradation, and vascular remodeling, testosterone may influence disease progression through androgen receptor–mediated pathways.

**Methods:**

A narrative literature review was conducted using PubMed/MEDLINE, EMBASE, and Google Scholar (August–September 2025). Studies examining testosterone, TRT, vascular inflammation, and aneurysm-related outcomes, including experimental, preclinical, and clinical studies, were included.

**Evidence synthesis:**

Available evidence is mostly mechanistic and preclinical. Animal studies indicate vascular bed-specific effects: physiological androgen signaling seems protective in abdominal aortic aneurysm models by reducing macrophage infiltration and inflammatory cytokine signaling, whereas exogenous or supraphysiologic testosterone may exacerbate oxidative stress and vascular injury in intracranial aneurysm models. Human data remain indirect, with no prospective studies specifically assessing aneurysm formation, growth, or rupture in TRT users. Large randomized trials and meta-analyses indicate cardiovascular safety comparable to placebo in appropriately selected patients, though higher rates of atrial fibrillation, pulmonary embolism, and erythrocytosis have been reported.

**Conclusion:**

Testosterone has context-, dose-, and vascular bed-specific effects on aneurysm biology. Until clinical studies on aneurysm outcomes are available, TRT should be individualized, dosed to meet physiological needs, and closely monitored for cardiovascular and vascular health. No evidence-based recommendation can currently be made to initiate, withhold, or modify TRT solely on the basis of aneurysm status. Multidisciplinary discussion and continued standard aneurysm surveillance are advisable for hypogonadal patients with known aneurysmal disease.

**Graphical Abstract:**

**Figure:**
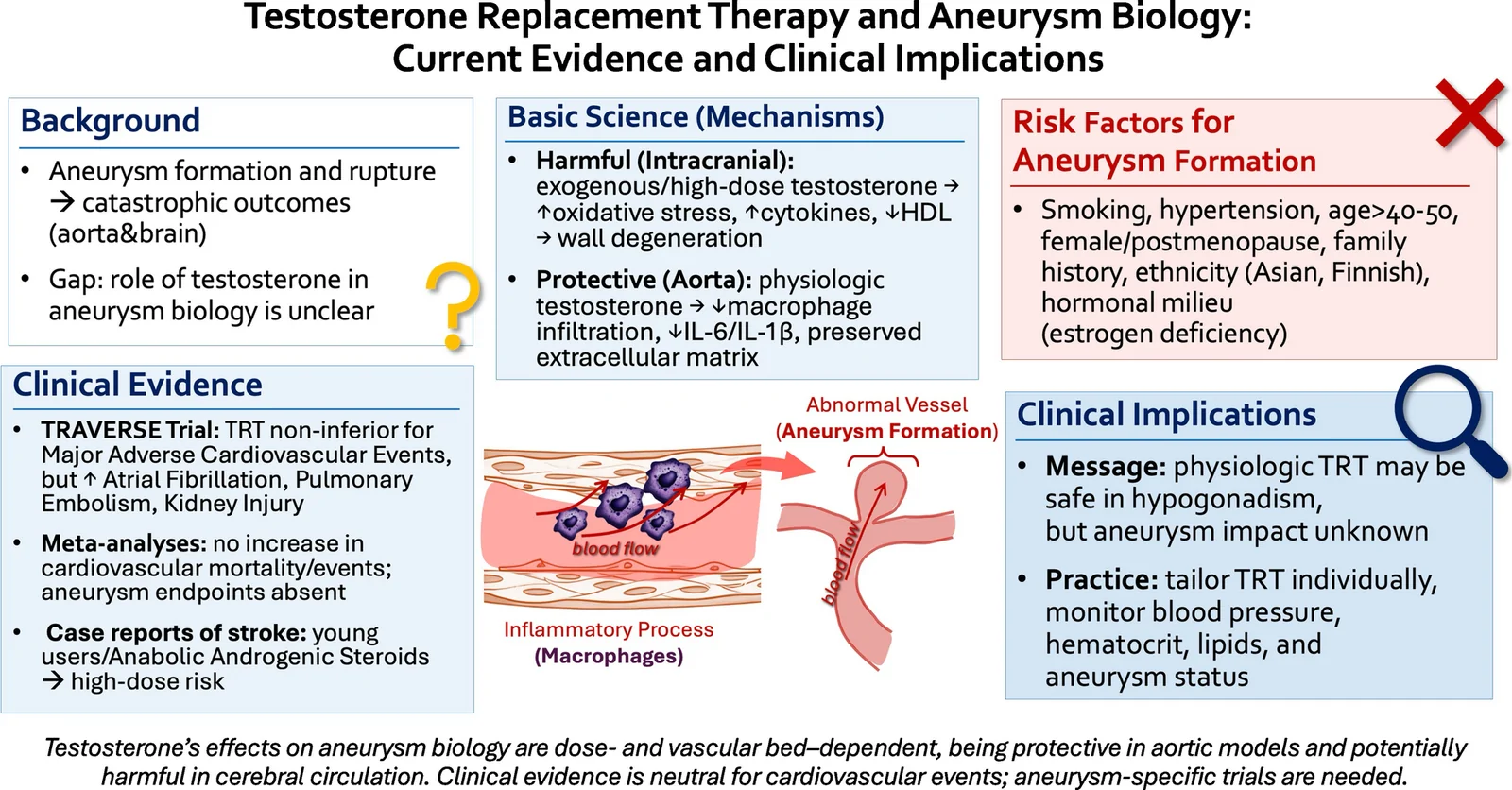

## Introduction

Aneurysms are pathological dilations of the arterial wall that can develop in multiple vascular territories, with the aorta and intracranial circulation the most clinically significant sites because rupture is associated with substantial morbidity and mortality [1]. The estimated lifetime prevalence of aortic aneurysms ranges from 1 to 8% in the general population, while unruptured intracranial aneurysms are found in approximately 2% to 3% of individuals. Both aortic and intracranial aneurysms have been reported in the same person in nearly 1% of cases [2, 3]. In particular, the initial mortality rate for intracranial aneurysm rupture approaches 50%, often resulting in long-term neurological impairments and disability [1, 4, 5].

The development and rupture of aneurysms are caused by a multifactorial process involving hemodynamic stress, genetic predisposition, environmental influences, and hormonal factors. Central to this process are chronic vascular inflammation and ongoing extracellular matrix degradation, which weaken the arterial wall and predispose it to dilate and rupture [1, 6]. Experimental models have underscored the important role of inflammatory cell infiltration—particularly macrophages—in vascular wall remodeling and aneurysm formation [7, 8]. In addition to inflammation, aneurysm growth and rupture are influenced by cardiovascular risk factors, such as hypertension, smoking, and dyslipidemia, as well as by biomechanical forces and vascular remodeling [6, 9].

Hormonal influences add another layer of complexity to aneurysm biology. Testosterone replacement therapy (TRT) is commonly prescribed for male hypogonadism and other conditions characterized by low serum testosterone. While TRT offers well-documented benefits—including enhanced sexual function, increased muscle mass, improved bone density, and better quality of life—its cardiovascular safety remains debated [10]. Recent epidemiological studies, mechanistic research, and meta-analyses suggest that testosterone may influence vascular remodeling, inflammatory signaling, and lipid metabolism, thereby affecting the risk of aneurysm formation and rupture [10]. Growing evidence points to sex hormones, particularly testosterone, as potential mediators of aneurysmal disease.

This review aims to address how TRT affects aneurysm formation, progression, and rupture risk across different vascular beds, synthesizing current mechanistic, preclinical, and clinical evidence on testosterone's effects on vascular remodeling, inflammatory signaling, oxidative stress, and extracellular matrix integrity. By integrating findings from experimental models and human studies, we aim to clarify the current evidence and its potential clinical implications for male patients with known or suspected aneurysmal disease.

## Methods

This article is a narrative literature review and was not conducted or reported as a systematic review. A comprehensive search of the *PubMed/MEDLINE*, *EMBASE*, and *Google Scholar* databases was conducted between August and September 2025. The following keywords, combined with Boolean operators, were used: *“testosterone”*, *“sex hormones”*, *“testosterone replacement therapy”*, *“aneurysm”*, *“intracranial aneurysm”*, *“aortic aneurysm”*, *“formation”*, *“growth”*, and *“rupture”*.

The search strategy was designed to capture a broad range of publications, including basic science studies, experimental animal models, clinical observational studies, meta-analyses, and narrative or systematic reviews. Studies were eligible if they addressed mechanisms of aneurysm formation and rupture, vascular remodeling, inflammation, or the effects of testosterone and related hormonal pathways on vascular biology. Both experimental and clinical evidence were considered relevant.

No restrictions were placed on publication date, but only articles published in English and available as full text were included. The reference lists of the included articles were also manually screened to identify additional relevant studies.

Given the descriptive nature of this review, no formal quality assessment or risk-of-bias scoring was performed. Extracted data were organized thematically into sections underscoring the role of TRT in aneurysm formation, progression, and rupture.

## Evidence synthesis

**Fig. 1:**
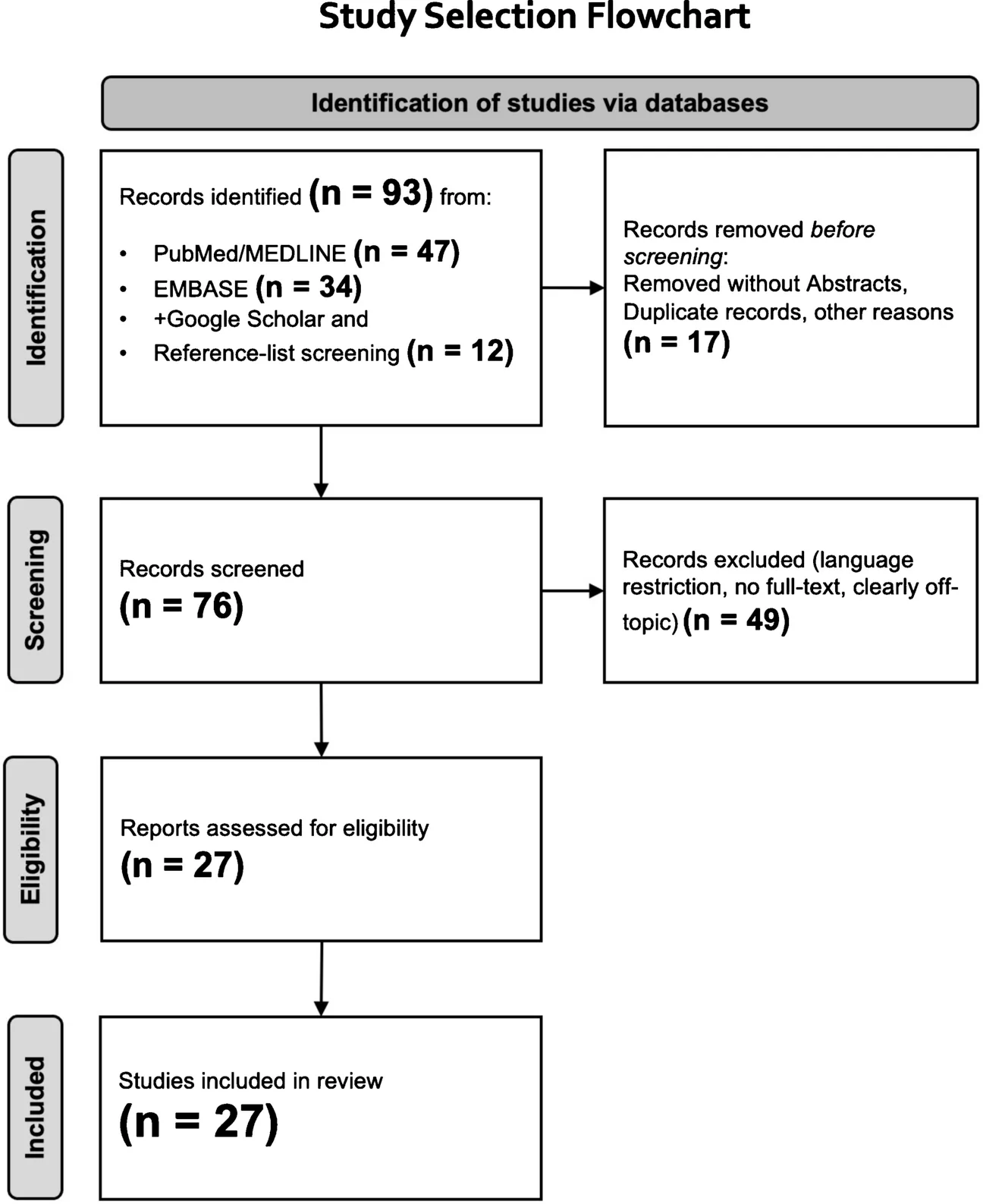
Study selection flowchart for the narrative literature review. A total of 93 records were identified: 47 from PubMed/MEDLINE, 34 from EMBASE, and 12 from Google Scholar and reference-list screening. Seventeen records were removed before screening (duplicates, no abstract, or other reasons), leaving 76 for screening. Records irrelevant to aneurysm formation, progression, rupture, vascular remodeling, inflammation, or hormonal signaling were excluded (n = 49), and 27 reports were assessed for eligibility and included in the qualitative narrative synthesis. Included articles were organized into four evidence domains: (1) mechanistic and vascular-biology studies; (2) animal models of aneurysm formation; (3) human clinical and epidemiologic data related to testosterone exposure; and (4) cardiovascular-safety studies of testosterone replacement therapy (TRT). This flowchart is provided for transparency and does not constitute or imply a systematic review, scoping review, or Preferred Reporting Items for Systematic Reviews and Meta-Analyses extension for Scoping Reviews (PRISMA-ScR)-compliant methodology

Using the predefined search strategy across PubMed/MEDLINE, EMBASE, and Google Scholar, we identified a limited but conceptually informative body of literature on testosterone and aneurysm biology. The database search retrieved 47 records from PubMed and 34 from EMBASE, while Google Scholar and reference-list screening yielded an additional 12 relevant articles (total identified: n = 93). Seventeen records were removed before screening (duplicates, no abstract, or other reasons), leaving 76 for screening. Records clearly irrelevant to aneurysm formation, progression, rupture, vascular remodeling, inflammation, or hormonal signaling were excluded (n = 49), leaving 27 articles for qualitative synthesis (see Fig. 1 for the study selection flowchart). These studies clustered into four evidence domains: (1) mechanistic and vascular-biology studies, (2) animal models of aneurysm formation, (3) human clinical and epidemiologic data related to testosterone exposure, and (4) cardiovascular-safety studies of TRT. Overall, the literature is weighted toward mechanistic and preclinical evidence, with very limited human data directly related to aneurysm-specific outcomes.

### Mechanistic background

**Fig. 2:**
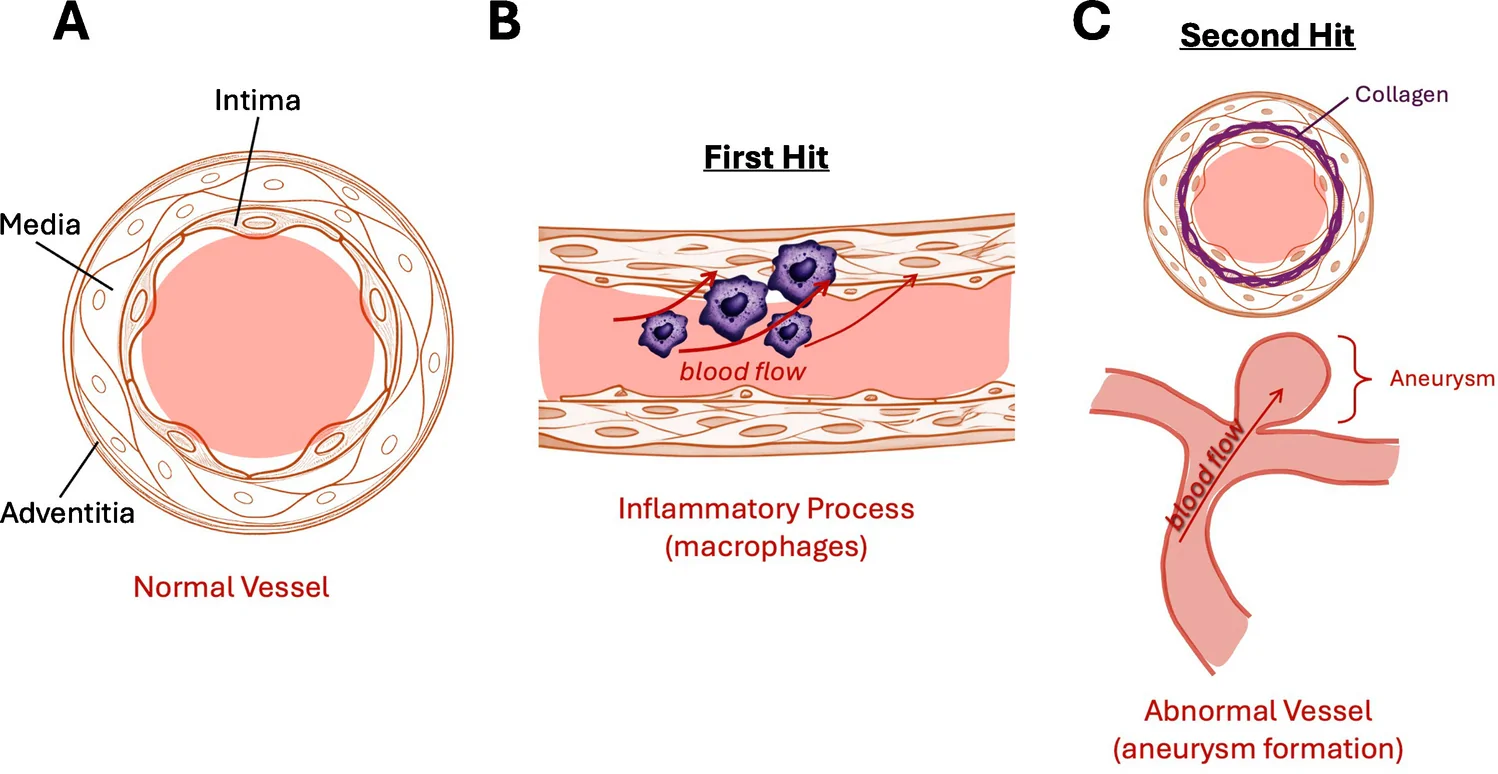
Schematic representation of the “two-hit hypothesis” for aneurysm formation. (**A**) Normal arterial wall with intact *intima*, *media*, and *adventitia*. (**B**) *First Hit*: Inflammatory cell infiltration (macrophages), vascular smooth muscle cell (VSMC) apoptosis, and disruption of the internal elastic lamina result in weakening of the intimal and medial layers. (**C**) *Second Hit*: maladaptive collagen remodeling and extracellular matrix (ECM) degradation under persistent hemodynamic stress, leading to vascular wall remodeling and aneurysm formation [6–8]. ***Abbreviations: VSMC****, vascular smooth muscle cell; ****ECM****, extracellular matrix*

Aneurysm development reflects the interaction of hemodynamic injury, vascular smooth muscle cell loss, inflammatory activation, and extracellular matrix failure [6, 11–13]. Disturbed shear stress and cyclic stretch initiate endothelial dysfunction and medial injury, while progressive degradation of elastin and collagen weakens the arterial wall and permits focal dilatation [12–16]. Within this framework, testosterone is relevant because androgen receptor signaling can alter endothelial nitric oxide bioavailability, macrophage activity, oxidative stress, and matrix turnover [17–19] **(**Fig. 2**)**.

### Inflammation

In both intracranial aneurysms and abdominal aortic aneurysms, macrophage recruitment is an early and persistent feature of wall injury [7, 8, 14]. Activated macrophages amplify nuclear factor kappa B (NF-κB)–dependent signaling, cytokine release, and prostaglandin pathways, thereby sustaining vascular inflammation and promoting aneurysm progression [14, 15]. This inflammatory background is important because several experimental studies suggest that testosterone can either dampen or intensify macrophage-driven responses depending on dose, formulation, and vascular bed [10, 17, 18].

### Impact on matrix metalloproteinases (MMPs)

Matrix metalloproteinases, particularly MMP-2 and MMP-9, are central mediators of elastin and collagen degradation in aneurysm walls [8, 15, 16]. Upstream inflammatory signaling through cyclooxygenase-2 (COX-2), microsomal prostaglandin E2 synthase-1 (mPGES-1), and prostaglandin E2 (PGE2), along with related cytokine cascades, increases MMP activity, linking immune-cell infiltration to structural failure [15, 16]. Experimentally, testosterone appears to act directly on this pathway: supraphysiologic or exogenous testosterone exposure upregulates MMP-2/MMP-9 expression and activity in vascular smooth muscle cells and macrophages, largely through androgen receptor-mediated amplification of NF-κB and COX-2/mPGES-1/PGE2 signaling, thereby accelerating elastin and collagen breakdown. By contrast, physiologic androgen signaling appears to restrain this cytokine-driven MMP induction, consistent with the protective pattern observed in abdominal aortic aneurysm models. Testosterone may therefore influence aneurysm growth, at least in part, through modulation of proteolytic activity rather than through a purely hemodynamic effect [15–18].

### Collagen dynamics and testosterone

Beyond inflammation, aneurysm progression depends on the balance among collagen synthesis, collagen remodeling, and smooth muscle cell survival [6, 11]. Physiologic androgen signaling may help preserve wall integrity by restraining inflammatory injury and apoptosis, whereas exogenous or supraphysiologic exposure may shift the balance toward oxidative stress, cell death, and maladaptive remodeling [10, 18]. Thus, testosterone should not be viewed as uniformly protective or harmful; its effect is likely contingent on biologic context and exposure profile [10, 17, 18].

### Regional variations in the vascular tree

The aorta and intracranial arteries differ substantially in wall architecture, vasa vasorum, baseline inflammatory milieu, and local flow conditions [12, 13]. These differences provide a plausible explanation for the divergent experimental findings across vascular beds: in abdominal aortic aneurysm models, physiologic androgen-receptor activity appears to limit macrophage-mediated cytokine signaling [18], whereas in intracranial aneurysm models, exogenous testosterone has been associated with oxidative stress, unfavorable lipid changes, and worsening vascular injury [10].

Taken together, the mechanistic literature supports a context-dependent model in which testosterone interacts with inflammation, matrix turnover, and regional vascular biology rather than exerting a single uniform effect across all aneurysm types [10, 17–19].

### Effects of testosterone on aneurysm biology

#### Animal studies

The available animal data are limited but informative, as summarized in Table 1 [10, 18, 20]. Broadly, abdominal aortic aneurysm models suggest a protective effect of physiologic androgen-receptor signaling, whereas intracranial aneurysm models suggest possible harm with exogenous testosterone exposure [10, 18].

**Table 1:** Animal studies evaluating testosterone and aneurysm-related vascular injury

Study/Model	Testosterone Exposure	Main Findings	Implications
*Murine AAA model*; Son et al. [18]	Castration, AR blockade, and physiologic testosterone replacement	Castration and AR blockade increased macrophage infiltration and IL-1beta/IL-6 signaling; physiologic replacement reduced inflammatory activity and aneurysm formation	Supports a protective effect of physiologic androgen signaling in the abdominal aorta
*Intracranial/cerebral vascular injury model*; Jin et al. [10]	Exogenous testosterone propionate	Higher oxidative stress, greater inflammatory cytokine expression, increased macrophage-related injury, and adverse lipid changes including lower HDL	Suggests that non-physiologic androgen exposure may aggravate cerebrovascular wall injury
*Ovariectomized rat aneurysm model*; Jamous et al. [20]	Estrogen replacement comparator (non-testosterone)	Hormone replacement reduced aneurysm formation and progression in an estrogen-deficient setting	Included as biologic context showing that sex-steroid tone influences aneurysm remodeling, although this is not a testosterone study

The available preclinical literature is limited and heterogeneous, but the studies are broadly useful, as they illustrate why testosterone must be considered in relation to dose, model, and vascular territory. Abbreviations: *AAA* Abdominal Aortic Aneurysm, *AR* Androgen Receptor, *HDL* High-Density Lipoprotein, *IL* Interleukin.

#### Human studies and clinical responses

Direct human evidence linking TRT to aneurysm formation, expansion, or rupture is currently absent [21–23]. No randomized trial or prospective cohort has used aneurysm-specific outcomes as a prespecified endpoint. The available human literature therefore informs this topic only indirectly, through cardiovascular safety trials of TRT, observational endocrine studies, and the broader sex hormone literature [19, 20, 22–24].

The Testosterone Replacement Therapy for Assessment of Long-term Vascular Events and Efficacy Response in Hypogonadal Men (TRAVERSE) trial and large meta-analytic datasets suggest that properly prescribed TRT is not associated with an excess of major adverse cardiovascular events in men with confirmed hypogonadism over short- to intermediate-term follow-up [22, 23]. However, these studies were not designed to evaluate aneurysm biology, included very few participants with known aneurysms, and cannot be interpreted as proof of aneurysm safety [22, 23].

Observational data contextualize these findings without providing confirmatory evidence for aneurysm-specific outcomes. Lower endogenous testosterone levels have been associated with greater abdominal aortic aneurysm prevalence in older men [19], whereas comparative sex-steroid literature in women suggests that hormonal milieu influences aneurysm susceptibility more broadly [20, 24]. These findings support biologic plausibility but remain insufficient to guide aneurysm-specific prescribing decisions **(**Table 2**)**.

**Table 2:** Summary of evidence on testosterone therapy, estrogen correlates, cardiovascular safety, and aneurysm biology

Evidence Source	Summary
Randomized Clinical Trials of TRT	Large randomized controlled trials (RCTs) (e.g., TRAVERSE) show testosterone replacement therapy (TRT) is non-inferior to placebo for major adverse cardiovascular events (MACE); increased atrial fibrillation (AF), pulmonary embolism (PE), and acute kidney injury (AKI) observed. No aneurysm endpoints included [22]
Meta-analyses of TRT	Pooled data (35 RCTs, ~ 5,200 men) show no increase in cardiovascular (CV) mortality or composite CV events. Rare aneurysm mentions only as incidental causes of death [23]
Illicit/supraphysiologic androgen use	Case reports and reviews suggest supraphysiologic TRT or anabolic–androgenic steroid (AAS) use is linked to thrombotic stroke and endothelial injury [21, 26]
Preclinical abdominal aortic aneurysm (AAA) models	Physiologic testosterone replacement after castration suppresses macrophage infiltration, interleukin (IL)−6/IL-1β signaling, and AAA formation [18]
Preclinical intracranial aneurysm (IA) models	Exogenous testosterone aggravates cerebral vascular injury, increasing reactive oxygen species (ROS), inflammatory cytokines, and reducing high-density lipoprotein (HDL) [10]
Female hormone studies	Estrogen deficiency increases risk of intracranial aneurysm and aneurysmal subarachnoid hemorrhage (aSAH); hormone replacement protective in both epidemiologic and animal studies [20, 24]
Regulatory perspective	U.S. Food and Drug Administration (FDA) updated labeling (2025): TRT neutral for MACE, but class warning for blood pressure and need for close monitoring [25]

*TRT* testosterone replacement therapy, *RCT* randomized controlled trial, *AF* atrial fibrillation, *PE* pulmonary embolism, *AKI* acute kidney injury, *CV* cardiovascular, *AAS* anabolic–androgenic steroids, *AAA* abdominal aortic aneurysm, *HDL* high-density lipoprotein, *aSAH* aneurysmal subarachnoid hemorrhage, *MACE* major adverse cardiovascular events, *FDA* Food and Drug Administration, *ROS* reactive oxygen species.

#### Potential adverse effects of testosterone replacement therapy

Even though aggregate cardiovascular-risk estimates are generally neutral in indicated TRT, clinically relevant safety signals remain important [22, 23]. Reported adverse effects include erythrocytosis, blood-pressure elevation, atrial fibrillation, pulmonary embolism, acute kidney injury, and possible prothrombotic effects related to increased blood viscosity and platelet activation [21, 22, 25]. In patients with aneurysmal disease, these issues matter because they may compound baseline vascular risk even when a direct aneurysm effect has not been demonstrated.

It is also essential to distinguish guideline-based TRT from anabolic–androgenic steroid misuse. Supraphysiologic androgen exposure, especially when combined with polypharmacy or non-medical use, has been linked more consistently to endothelial injury, thrombosis, cardiomyopathy, and accelerated atherosclerosis [21, 26]. These observations should not be conflated with standard TRT, but they underscore that dose and exposure pattern are critical determinants of vascular toxicity [21, 26].

**Fig. 3:**
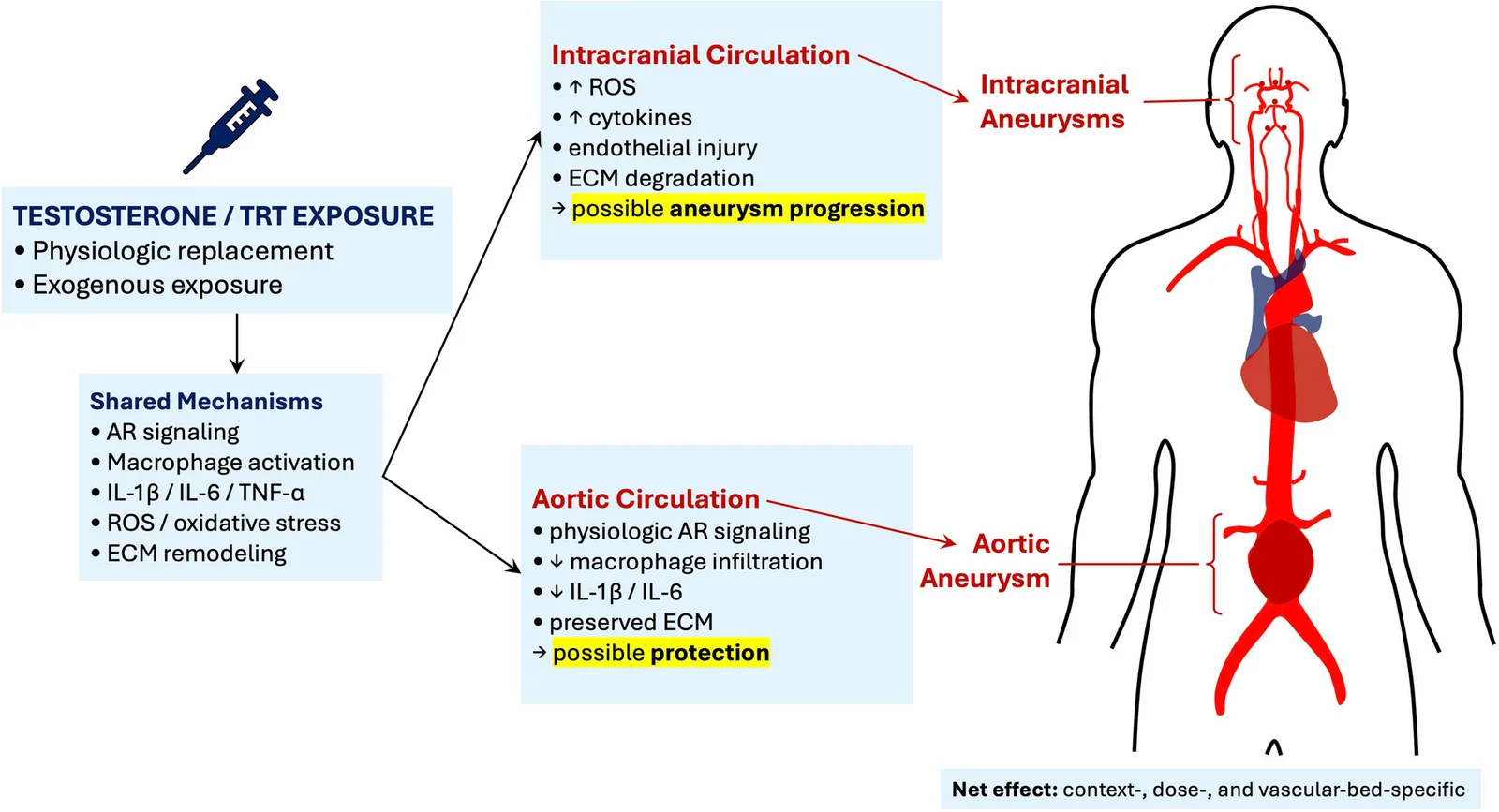
Central mechanistic model of testosterone replacement therapy (TRT) in aneurysm biology. Testosterone influences aneurysm-related vascular remodeling through androgen receptor (AR) signaling, modulation of macrophage-driven inflammation, oxidative stress (reactive oxygen species [ROS]), endothelial dysfunction, lipid metabolism, and extracellular matrix (ECM) degradation via matrix metalloproteinases (MMPs). Available preclinical evidence suggests vascular-bed-specific effects: physiologic androgen signaling may reduce inflammatory remodeling in abdominal aortic aneurysm (AAA) models, while exogenous or non-physiologic testosterone exposure may promote oxidative and inflammatory wall injury in intracranial aneurysm (IA) models. Overall, the vascular effects of testosterone appear to depend on the context, dose, and tissue [10, 13, 15, 17–20, 22–24]. ***Abbreviations: AR****, androgen receptor; ****ROS****, reactive oxygen species; ****ECM****, extracellular matrix; ****MMP****, matrix metalloproteinase; ****NF-κB****, nuclear factor kappa B; ****NO****, nitric oxide; ****HDL****, high-density lipoprotein; ****AAA****, abdominal aortic aneurysm; ****IA****, intracranial aneurysm; ****TRT****, testosterone replacement therapy*

Regulatory agencies have responded to this mixed evidence by maintaining close monitoring recommendations for testosterone products, including blood-pressure surveillance and careful patient selection [25]. From an aneurysm perspective, the most defensible conclusion is that the absence of direct evidence should be interpreted as uncertainty rather than reassurance [27] **(**Fig. 3**)**.

## Discussion

The revised synthesis clarifies that the strongest evidence in this field is mechanistic, not clinical. Testosterone appears capable of modulating several pathways that are central to aneurysm biology, including macrophage-driven inflammation, MMP activity, oxidative stress, endothelial dysfunction, and matrix remodeling [10, 14–19]. However, the direction of effect is not uniform. Experimental abdominal aortic aneurysm data favor a protective role for physiologic androgen-receptor signaling [18], whereas intracranial models raise concern about potential injury under exogenous or non-physiologic exposure [10].

This distinction has practical implications for interpretation. It would be inappropriate to extrapolate the apparent cardiovascular neutrality of TRT to aneurysm neutrality, and equally inappropriate to infer harm from isolated preclinical models without clinical corroboration [22, 23]. The main message of the present review is therefore not that TRT is either safe or unsafe for aneurysm patients, but that the available evidence is biologically compelling, clinically incomplete, and strongly dependent on vascular territory and exposure profile [10, 18, 22, 23].

### Clinical implications

From a sex-differences perspective, the differential effects of the hormonal milieu on aneurysm biology represent an important emerging frontier. Available data on estrogen’s protective role in intracranial aneurysm susceptibility, together with the epidemiologic observation that aneurysmal subarachnoid hemorrhage (aSAH) risk increases postmenopausally in women, suggest that the estrogen-to-testosterone ratio — rather than any single hormone in isolation — may be a key determinant of vascular risk [17, 20, 24]. Future research incorporating sex-stratified analyses would be valuable.

For men with confirmed hypogonadism and a known aortic or intracranial aneurysm, the following guidance can be offered on the basis of the current, necessarily indirect, evidence: It should be noted explicitly that none of these recommendations derive from prospective studies with aneurysm-specific endpoints; they represent cautious expert interpretation of mechanistic and cardiovascular safety data in the aneurysm context [10, 18, 22, 23]:

1. TRT should not be reflexively withheld solely because of an aneurysm diagnosis, as no direct evidence demonstrates harm in this population [22, 23].
2. Physiologic testosterone levels should be targeted; supraphysiologic exposure should be avoided because preclinical data link non-physiologic androgen levels to oxidative stress and vascular injury in intracranial models [10, 18].
3. Known cardiovascular risk factors modifiable by TRT — erythrocytosis (hematocrit), blood pressure, and lipid profile — should be actively monitored and managed throughout treatment, as these factors may compound aneurysm-related vascular risk even in the absence of a direct aneurysm effect [21, 22, 25].
4. Aneurysm surveillance should continue according to established location- and size-based protocols, regardless of TRT status, because no evidence supports either reducing or intensifying surveillance based solely on TRT use [11].
5. In patients with significant aneurysmal disease, a multidisciplinary discussion involving vascular surgery or neurosurgery and endocrinology is advisable before initiating or continuing TRT to ensure that shared decision-making incorporates both hypogonadism management and aneurysm risk [22, 25].

Future work should prioritize prospective cohorts and pragmatic studies with aneurysm-specific endpoints, stratified by vascular bed, formulation, and achieved testosterone levels.

### Limitations

This review has limitations. The available evidence is predominantly mechanistic and preclinical, with very limited human data directly addressing aneurysm-specific outcomes in patients receiving TRT. The included studies are heterogeneous with respect to experimental models, vascular territories, testosterone formulations, dosing regimens, and outcome measures, limiting direct comparisons and generalizability. Because this was a narrative literature review without a formal risk-of-bias assessment or meta-analysis, the conclusions should be viewed as hypothesis-generating rather than definitive. The lack of prospective clinical studies on aneurysm formation, progression, or rupture under TRT remains a significant evidence gap that limits firm clinical recommendations.

## Conclusion

In conclusion, three principal messages emerge from this review: *First*, testosterone’s vascular effects are vascular-bed-specific and dose-dependent: physiologic androgen receptor signaling appears to limit macrophage-driven inflammation and may reduce the risk of abdominal aortic aneurysm formation, whereas non-physiologic or supraphysiologic exposure is associated with increased oxidative stress and inflammatory injury in intracranial aneurysm models. *Second*, no clinical trial has assessed aneurysm outcomes in TRT users; the cardiovascular neutrality demonstrated in large randomized trials cannot be extrapolated to aneurysm safety, and TRT cannot be considered proven safe or unsafe in this population on current evidence. *Third*, for hypogonadal patients with known aneurysmal disease, the most defensible clinical approach — pending prospective data — is physiologic dosing, active management of erythrocytosis, blood pressure, and lipids, continuation of standard aneurysm surveillance, and multidisciplinary discussion. Definitive guidance will require prospective studies of aneurysm formation, growth, and rupture in TRT users, combining mechanistic biomarkers and imaging endpoints, stratified by vascular bed and testosterone formulation.

## Data Availability

All data underlying this study are derived from previously published articles indexed in PubMed/MEDLINE, EMBASE, and Google Scholar. No new datasets were generated or analyzed in this review. References to all included are provided in the manuscript.

## Declarations

### AI declaration

Generative Artificial Intelligence was not used in study design, data analysis, interpretation, or the generation of scientific content. An AI-based language tool was used solely to check grammar and improve English readability; the authors reviewed all AI-assisted edits and take full responsibility for the content of this manuscript.
